# Exploring the Role of Matrix Metalloproteinases as Biomarkers in Sporadic Lymphangioleiomyomatosis and Tuberous Sclerosis Complex. A Pilot Study

**DOI:** 10.3389/fmed.2021.605909

**Published:** 2021-04-26

**Authors:** Silvia Terraneo, Elena Lesma, Silvia Ancona, Gianluca Imeri, Giuseppina Palumbo, Olga Torre, Lisa Giuliani, Stefano Centanni, Angela Peron, Silvia Tresoldi, Paola Cetrangolo, Fabiano Di Marco

**Affiliations:** ^1^Department of Health Sciences, Università Degli Studi di Milano, Milan, Italy; ^2^Respiratory Unit, Azienda Socio Sanitaria Territoriale Santi Paolo e Carlo, Milan, Italy; ^3^Laboratory of Pharmacology, Department of Health Sciences, Università Degli Studi di Milano, Milan, Italy; ^4^Respiratory Unit, Azienda Socio Sanitaria Territoriale - Papa Giovanni XXIII Hospital, Bergamo, Italy; ^5^Human Pathology and Medical Genetics, Azienda Socio Sanitaria Territoriale Santi Paolo e Carlo, San Paolo Hospital, Milan, Italy; ^6^Child Neuropsychiatry Unit - Epilepsy Center, Department of Health Sciences, Azienda Socio Sanitaria Territoriale Santi Paolo e Carlo, San Paolo Hospital, Università Degli Studi di Milano, Milan, Italy; ^7^Division of Medical Genetics, Department of Pediatrics, University of Utah School of Medicine, Salt Lake City, UT, United States; ^8^Radiology Unit – Azienda Socio Sanitaria Territoriale Santi Paolo e Carlo, San Paolo Hospital, Milan, Italy

**Keywords:** lymphangiomeiomyomatosis, tuberous sclerosis complex, biomarkers, matrix metalloproteinases, vascular endothelial growth factor

## Abstract

**Background:** Lymphangioleiomyomatosis can develop in a sporadic form (S-LAM) or in women with tuberous sclerosis complex (TSC). The matrix metalloproteinases (MMPs) are extracellular matrix-degrading enzymes potentially involved in cystic lung destruction, and in the process of migration of LAM cells. The aim of the study was to explore the role of MMP-2 and MMP-7, such as vascular endothelial growth factor (VEGF) -C and -D in women with LAM, including patients with minor pulmonary disease (i.e., <10 lung cysts), and TSC with or without LAM.

**Methods:** We evaluated 50 patients: 13 individuals affected by S-LAM, 20 with TSC-LAM, of whom six with minor pulmonary disease, and 17 with TSC without pulmonary involvement. Sixteen healthy women were used as controls.

**Results:** MMP-2 resulted higher in LAM compared to healthy volunteers, and TSC patients (*p* = 0.040). MMP-7 was higher in TSC-LAM patient, with even greater values in patients with TSC-LAM minor pulmonary disease, than in S-LAM patients, and in controls (*p* = 0.001). VEGF-D level was lower than 800 pg/mL in all healthy controls and resulted higher in S-LAM and TSC-LAM than in TSC patients and controls (*p* < 0.001). VEGF-C values were not statistically different in the study population (*p* = 0.354). The area under ROC curves (AUCs) of MMP-2, and MMP-7 for predicting LAM diagnosis were of 0.756 ± 0.079 (*p* = 0.004), and 0.828 ± 0.060 (*p* < 0.001), respectively. Considering only patients with TSC, the AUCs for MMP-2, and MMP-7 in predicting LAM were 0.694 ± 0.088 (*p* = 0.044), and 0.713 ± 0.090 (*p* = 0.027), respectively.

**Conclusions:** Our data suggest that MMP-2 and MMP-7 could be promising biomarkers for LAM diagnosis.

## Introduction

Lymphangioleiomyiomatosis (LAM) is a rare progressive cystic lung disease affecting mostly women in the childbearing age (1). LAM, seen either as a sporadic form (S-LAM) or as a manifestation of tuberous sclerosis complex (TSC-LAM), is clinically characterized by progressive dyspnea, recurrent pneumothorax, and chylous pleural effusions (2). The extent of pulmonary cystic involvement in both forms of LAM varies from minor disease, with the identification of few cysts on chest CT scan, to diffuse destruction of the lungs (3). In most cases LAM progresses into respiratory failure (1, 2), but in some patients the disease can remain asymptomatic or even undiagnosed (4).

The cystic modification of the lung parenchyma is thought to be caused by the proliferation of abnormal smooth muscle-like cell called “LAM cells” in the pulmonary interstitial and along the axial lymphatics in the thorax and abdomen. LAM cells exhibit a unique immunophenotype as they express desmin and actin and gp100 and react with HMB-45 antibody (5). The mechanism of cysts development by LAM cells is not completely understood; however different mechanisms seem to contribute to lung destruction (6). LAM cells can express vascular endothelial growth factor (VEGF), a lymphangiogenic protein. Two isoforms of VEGF have been isolated in LAM patients: VEGF-C, which is expressed in lung tissues, and VEGF-D, which was isolated in the serum of patients with LAM (7, 8). VEGF is able to recruit lymphatic endothelial cells, driving the formation of lymphatic vascular channels. This abnormal lymphangiogenesis leads to vascular and airways obstruction and consequent development of air cystic lesions in the pulmonary parenchyma (6). Serum levels of VEGF-D are high in most patients with LAM and—in association with the characteristic cystic images seen on CT scan—could be diagnostic for LAM, thus avoiding invasive testing such as lung biopsy (9, 10). In patients with LAM, VEGF-D serum level correlates with disease severity, namely chylous effusions and/or lymphatic involvement (11, 12), lung function at presentation and rate of disease progression (13) systemic involvement in patients with TSC (14), and pulmonary functional impairment (15, 16). Patients with high VEGF-D serum level are more likely to respond to treatment with sirolimus than patients with low VEGF-D serum level (17).

Immunochemical studies demonstrated an over expression of matrix metalloproteinases (MMPs) and MMPs inducers in lung tissues of patients with LAM, and a tissue paucity of MMPs tissue inhibitor (TIMP)-1 (18). MMPs are zinc-dependent endopeptidases that are active in tissue remodeling by degrading extracellular matrix collagen and elastine (19). Degradation of elastic fibers in areas of smooth muscle proliferation was found in lung biopsy specimens of patients with LAM (20). Hence, MMPs are suggested to be involved in lung tissue destruction and cysts formation in LAM. Odajima et al. demonstrated that serum levels of MMP-9 isoform were higher in LAM patients compared to healthy controls (21), suggesting a possible involvement of MMP-9 in LAM development. However, MMP-9 levels cannot be correlated with LAM severity, evaluated as cystic parenchymal involvement quantified by lung high resolution computed tomography (HRCT) (22). MMP-2 is the most expressed MMP isoform in bronchiolar and vascular smooth muscle cells, in basement membranes of LAM tissue, and in overlying epithelial cells (18). However, some studies suggest that serum MMP-2 levels cannot be correlated with the extent of pulmonary cystic involvement. MMP-7 is one of the most important MMPs in elastin turnover (23). Barnes et al. demonstrated that the invasion of tuberin-null cells might be mediated by MMP-7 (matrilysin), a component of cell invasion, in a TSC model and in LAM tissues (24). However, data about serum level of MMP-7 in patients with LAM are lacking.

Thus, the aim of the present study was to explore the role of MMP-2 and MMP-7 as biomarkers in a cohort of patients with LAM, both S-LAM and TSC-LAM, and in a subgroup of TSC patients with minor cystic parenchymal involvement. An association of such biomarkers, including VEGF-D and -C, with systemic involvement was also explored.

## Methods

### Study Design and Population

We performed a cohort study involving adult female outpatients affected by S-LAM and/or TSC followed (1) at the pulmonary clinic (S-LAM, TSC-LAM) and (2) at the Tuberous Sclerosis Center (TSC) of San Paolo Hospital, Milan, Italy, from January 2014 to December 2017.

The diagnoses of both LAM and TSC were based on previously published guidelines (25–27). A multidisciplinary evaluation was performed according to the international guidelines as previously reported (14) in the following patients: (1) individuals with a diagnosis of TSC; (2) patients with diagnosis of LAM who were evaluated in the Pulmonology clinic to rule out a form of TSC-related LAM. Imaging was performed when needed (i.e., abdominal magnetic resonance imaging (MRI) or ultrasound for angiomyolipoma (AML) diagnosis and follow up, MRI of the brain for neurologic involvement). A clinical and radiological follow-up plan was established on a case-by-case basis and according to the international guidelines (27). Thoracic imaging with lung HRCT was requested during the first pulmonary evaluation and repeated in case of respiratory symptoms. A peripheral blood sample for the evaluation of MMP-2, MMP-7, VEGF-C and VEGF-D was obtained during one of the scheduled pulmonary evaluations in clinic. Clinical and radiological data were collected within 3 months after the serum biomarkers results. All patients were in an observational cohort with Hospital Ethics Committee approval. All patients or relatives, in case of patients with intellectual disability, provided informed consent.

### Quantification of Serum VEGF-D, VEGF-C, MMP-2, and MMP-7

Peripheral blood was collected in serum separator tubes, allowed to clot for 30 min at 4°C, centrifuged at 1000 × g for 15 min. Serum was aliquoted and stored at −80°C. Serum VEGF-D, VEGF-C, MMP-2, and MMP-7 were measured using Quantikine Human Immunoassays (R&D Systems; Minneapolis, MN) according to the manufacturers' instruction.

### Pulmonary Involvement Assessment

Spirometry, body pletismography and lung diffusion tests (Platinum Elite™ MGC Diagnostic, USA) were performed according to the ATS/ERS guidelines (28, 29). Dyspnea was investigated throughout the Italian version of the modified Medical Resource Council (MRC) scale (30). The six-minute walk test (6MWT) was performed along a flat, straight, 30-m walking course supervised by a well-trained researcher according to the ATS guidelines (31).

### Genetic Analyses

Qiamp DNA blood mini DNA kit (Qiagen, Germany) was employed to extract DNA from peripheral lymphocytes (Qiagen, Germany). *TSC1* and *TSC2* exons from genomic DNAs were amplified by means of standard polymerase chain reaction (PCR) using previously described primers (32). Pathogenic variants were detected by denaturing high-performance liquid chromatography (DHPLC) (Transgenomic, Crewe, UK). The products showing variant DHPLC melt profiles were directly sequenced using a BigDye terminator cycle sequencing kit (Applied Biosystems), and the results were analyzed using sequence analysis 3.4.1 software (ABI 3130, Applied Biosystem). The sequencing reactions for identified mutations were repeated. Patients that had negative investigations for DHPLC were evaluated with Multiple Ligation-dependent Probe Amplification test for *TSC1* (P124-MRC-Holland) and *TSC2* (P046-MRC-Holland) as previously described (33). Patients in whom genetic analysis was inconclusive were classified as having no mutation identified (NMI) after conventional genetic testing, as Next Generation Sequencing for TSC was not yet available in the laboratory at the time of the study.

### Image Analysis

All chest CT examinations (performed using a LightSpeed VCT—GEHealthcare, Milwaukee, WI-−64 slices scanner) were evaluated by a radiologist experienced in LAM, blinded to other researchers. The severity of cystic lung disease was graded according to a visual quantitative grading system (34). The lung involvement was classified as minimal pulmonary disease (Grade 0) if patients showed <10 lung cysts (25). If more than 10 cysts were identified then the extent of disease was graded as “mild” (Grade 1) if less than one third of the lung was involved; “moderate” (Grade 2) if one to two thirds of the lungs resulted involved; and “severe” (Grade 3) if cysts involved more than two third of the lungs. The presence of parenchymal nodules compatible with multifocal micronodular pneumocyte hyperplasia (MMPH) was also evaluated. Lymphatic involvement was considered in the presence of mediastinal lymph node enlargement, and/or pleural effusion attributed to chylothorax and/or lymphangioleiomyomas. Pneumothorax, when detected, was reported, too.

### Statistical Analysis

The results are shown as median and interquartile range (IQR), unless otherwise stated. Lilliefors corrected K-S test was performed before the data analysis in order to examine the distribution of the residuals of the parametric tests. For comparisons between patients, the Wilcoxon rank-sum test, Mann-Whitney test, and Kruskal-Wallis test were used, as appropriate. To detect the optimal cut off point at which the sensitivity and specificity of every biomarker were maximized, we developed a receiver operating characteristic (ROC) curve. Since there were no previous works that have analyzed the link between serum MMP-2 and MMP-7 and systemic involvement in LAM or TSC, we divided the patients (both with S-LAM and TSC) into two groups based on the 50th percentile of the distribution of the biomarker. All tests were two-sided, and *p* < 0.05 were considered statistically significant. Statistical tests were performed using the Statistical Package for Social Sciences (version 21.0; SPSS, Chicago, IL) and GraphPad Prism 7 (GraphPad Software, San Diego, California, USA).

## Results

Data from 50 patients were available for the analysis: 13 patients (20%) were affected by S-LAM, and 37 (56%) by TSC. Of these, 14 (38%) had TSC-LAM, six TSC-LAM minor pulmonary disease, and 17 TSC without LAM. Sixteen healthy women were used as controls. S-LAM was histologically confirmed in nine patients (by pulmonary biopsy in seven patients, by biopsy of abdominal lymphangioma in one patient and by identification of LAM cells in the chylous effusion in one patient). In four patients with S-LAM the diagnosis was clinically confirmed by the simultaneous presence of characteristic chest HRCT and renal AMLs. For patients with TSC the disease was clinically confirmed on the basis characteristic or compatible chest HRCT. All patients affected with LAM were taking standard inhalation therapy (long acting B_2_-agonists and long acting muscarinic agents). None was treated with Sirolimus or Everolimus at the time of biomarkers analysis. Demographic, clinical and genetic characteristics of the study population are reported in Table 1. There were no differences between groups in age at LAM diagnosis. One patient with S-LAM, three patients with TSC-LAM, three healthy, controls and one patient with TSC were menopausal, without any difference between groups (*p* = 0.429). All patients with a TSC-LAM minor pulmonary disease showed multifocal micronodular pneumocyte hyperplasia (MMPH) at chest HRCT. Dyspnea was common in patients with LAM, and almost half of the individuals with sporadic LAM reported the symptoms, whereas no patients had respiratory failure (Table 1). History of pneumothorax was reported in patients with LAM, including patients with minor pulmonary disease, while patients with S-LAM tended to have more frequently chylothorax than other patients (*p* = 0.063). Individuals with TSC-LAM showed more frequently renal AMLs than patients with S-LAM (*p* = 0.001). No differences were observed regarding the size of AMLs or ymphatic involvement, (such as lymphocele or mediastinal lymphadenopathy), hepatic AMLs, or *TSC1/TSC2* pathogenic variants between the groups. The analysis of functional data show that patients with S-LAM tended to lower FEV1 and significantly lower DLCO (mL/min/mmHg) than other subjects (Table 1).

**Table 1:** Demographic and clinical characteristics of the population in analysis.

	**S-LAM *N* = 13**	**TSC-LAM *N* = 14**	**TSC-LAM minor pulmonary disease *N* = 6**	**TSC *N* = 17**	**HEALTHY *N* = 16**	***p***
Age^*^, yrs, median (IQR)	36 (31–43)	36 (30–50)	34 (24–63)	32 (24–42)	36 (28–49)	0.831
Age at LAM diagnosis, yrs, median (IQR)	35 (29–44)	33 (27–45)	29 (22–61)	–	–	0.930
Smoking history (yes/no/ex), %	9/27/64	14/21/64	17/1/67	6/94/0	0/100/0	0.309
**Pulmonary involvement and symptoms**						
MMPH, *n* (%)^**^	–	8 (57)	6 (100)	10 (59)	–	0.068
Dyspnea^***^, *n* (%)	5 (38)	3 (21)	2 (33)	5 (29)	–	0.637
SpO2 <90% during 6mWT, *n* (%)	4 (36)	3 (21)	0 (0)	2 (12)	–	0.235
Pneumothorax, *n* (%)	4 (36)	3 (21)	1 (17)	0 (0)	–	0.082
Respiratory failure, *n* (%)	0 (0)	0 (0)	0 (0)	0 (0)	–	–
Chylotorax, *n* (%)	3 (27)	1 (7)	0 (0)	0 (0)	–	0.063
Lymphocele, *n* (%)	2 (18)	1 (7)	1 (6)	0 (0)	–	0.0592
Mediastinal lymphadenopathy, *n* (%)	1 (9)	2 (17)	1 (8)	1 (16)	–	0.896
**Abdominal involvement**						
Renal AMLs, *n* (%)	4 (36)	14 (100)	6 (100)	11 (65)	–	**0.001**
AMLs size (> 3 cm), *n* (%)	2 (11)	10 (53)	4 (21)	3 (16)	–	0.368
Hepatic AMLs, *n* (%)	1 (9)	6 (43)	5 (24)	1 (17)	–	0.268
Genotype^**^						
*TSC1, n* (%)	–	3 (21)	2 (33)	9 (56)	–	0.189
*TSC2, n* (%)	–	7 (50)	4 (67)	5 (31)	–	
NMI, *n* (%)	–	4 (29)	0 (0)	2 (13)	–	
Systemic TSC involvement^**^						
Renal tumor, *n* (%)	–	1 (8)	0 (0)	4 (24)	–	0.258
Cutaneous involvement, *n* (%)	–	13 (100)	6 (100)	17 (100)	–	>0.999
Epilepsy, *n* (%)	–	3 (23)	4 (67)	12 (71)	–	**0.027**
Cortical tubers, *n* (%)	–	12 (92)	6 (100)	15 (88)	–	0.665
**Pulmonary function, median (IQR) or median [min–max]**						
FVC (L)	2.81 (2.39–3.49)	3.64 (3.13–3.94)	2.96 [2.88–4.16]	3.53 (3.09–4.00)	–	0.264
FVC (%)	82 (75–97)	99 (90–110)	108 [67–122]	98 (86–109)	–	0.174
FEV1 (L)	2.36 (1.79–2.84)	3.02 (2.52–3.21)	2.57 [2.27–3.57]	3.14 (2.74–3.38)	–	0.063
FEV1 (%)	86 (64–97)	100 (85–110)	98 [69–99]	98 (89–113)	–	0.080
FEV1/FVC (%)	100 (84–106)	100 (92–105)	99 [99–102]	101 (100–103)	–	0.754
DLCO (mL/min/mmHg)	13.48 (10.39–16.31)	20.98 (17.66–23.05)	21.03 [13.71–23.62]	21.87 (19.57–22.90)	–	0.002
DLCO (%)	53 (41–67)	79 (65–81)	70 (60–91)	83 (74–90)	–	0.002
KCO (mL/min/mmHg/L)	3.47 (2.27–4.10)	4.11 (3.55–4.92)	5.07 [3.66–5.55]	4.56 (4.08–5.53)	–	0.015
KCO (%)	66 (44–88)	73–(67–78)	81 [80–99]	78 (76–95)	–	0.043
**Radiological involvement**^**§**^						
Grade 0, *n* (%)	0 (0)	0 (0)	6 (100)	–	–	<0.001
Grade 1 *n* (%)	2 (22)	10 (77)	0 (0)	–	–	
Grade 2, *n* (%)	4 (44)	1 (8)	0 (0)	–	–	
Grade 3, *n* (%)	3 (33)	2 (15)	0 (0)	–	–	

* *referred to age at time of blood sample evaluation*;

** *percentage are referred to total patients with TSC*;

*** *mMRC>1*;

§ *percentage are referred to patients with radiological analysis available*.

*IQR, interquartile range; MMPH, multifocal micronodular pneumocyte hyperplasia; TSC1/2/NMI, pathogenic variant of TSC1 or TSC2/no mutation identified; AML: angiomyplipoma; 6mWT, six minutes walking test. p < 0.050 in bold*.

### Distribution of Serum Biomarkers Between Groups

**Figure 1:**
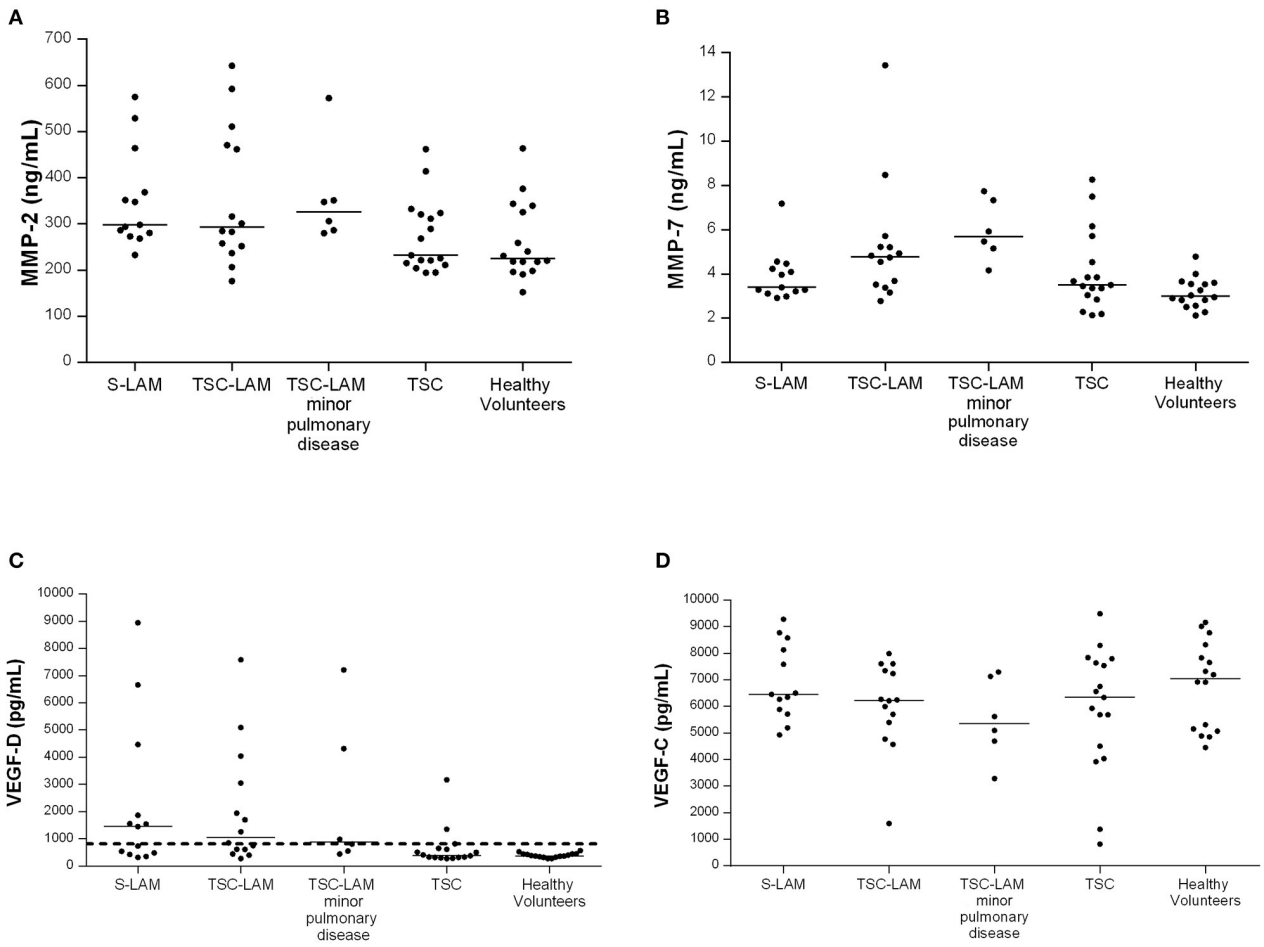
Distribution of MMP-2 **(A)** *p* = 0.040, MMP-7 **(B)** *p* = 0.001, VEGF-D **(C)** *p* < 0.001, VEGF-C **(D)** *p* = 0.354, in the study population (p refers to ANOVA); LAM, lymphangioleiomyomatosis; TSC, tuberous sclerosis complex; minor pulmonary disease: patients with <10 cysts identified at chest CT scan; bar represents the median value, dashed line represents VEGF-D value of 800 pg/mL.

As shown in Figure 1, serum MMP-2 was higher in the S-LAM group [median value: 298 ng/ml, range 277–416 ng/ml], and in the TSC-LAM patients (median value: 293 ng/ml, range 248–480 ng/ml) compared to healthy volunteers (median value: 225 ng/ml, range 203–336 ng/ml) and TSC patients (median value: 232 ng/ml, range 213–323 ng/ml, *p* = 0.040 of ANOVA). Serum MMP-7 was higher in TSC-LAM patients (median value: 4.78 ng/ml, range 3.5–5.3 ng/ml) with even greater values in patients with TSC-LAM minimal pulmonary disease (median value: 5.69 ng/ml, range: 4.90–7.43 ng/ml) than in S-LAM patients (median: 3.39 ng/ml, range: 3.16–4.35) and in controls (median: 2.99 ng/ml, range 2.62–3.59 ng/ml, *p* = 0.001 of ANOVA). There was no difference in genotype, lymphatic involvement, abdominal and systemic involvement between patients with MMP2 or MMP7 lower or higher than the 50th percentile except for a higher frequency of cortical tubers in TSC patients with MMP-7 >50th percentile (100 vs. 77%, *p* = 0.040).

Serum VEGF-D level was lower than the diagnostic threshold of 800 pg/mL in all healthy controls and resulted higher in the S-LAM group (median value: 1,456 pg/ml, range 457–3,167 pg/ml) and TSC-LAM group (median value: 1,057 pg/ml, IQR range 574–3,302 pg/ml) than in TSC patients (median value: 396 pg/ml, range 322–646 pg/ml) and controls (median value: 378 pg/ml range 335–444 pg/ml, *p* < 0.001 of ANOVA) (Figure 1). Patients with a VEGF-D higher than 800 pg/mL were significantly younger (median age 32 yrs, IQR 26–37 vs. 37 yrs, IQR 30–49; *p* = 0.005); they were diagnosed with LAM at a younger age (median age 32 yrs, IQR 24–36 vs. 44 yrs, IQR 29–56; *p* = 0.072), and had more frequent chylothoraces (19 vs. 0%, *p* = 0.034). They had more frequently pathogenic variants in *TSC2* (71 vs. 24%, *p* = 0.004), larger AMLs (>3 cm 13.87 vs. 6.30% *p* = 0.002), and more frequently retinal hamartomas than patients with low VEGF-D (6.43 vs. 2.10%; *p* = 0.042).

VEGF-C in serum was slightly but not significantly lower in S-LAM patients (median value: 6,453 pg/ml, range 585–8,325 pg/ml), TSC-LAM patients (median value: 6,230 pg/ml, range 5,240–7,411 pg/ml), and TSC patients (median value: 6,338 pg/ml, range 4,271–7,718 pg/ml) than controls (median value 7,058 pg/ml, range 5,093–8,198 pg/ml, *p* = 0.354 of ANOVA).

### Diagnostic Yield of Serum Biomarkers for LAM Diagnosis

**Figure 2:**
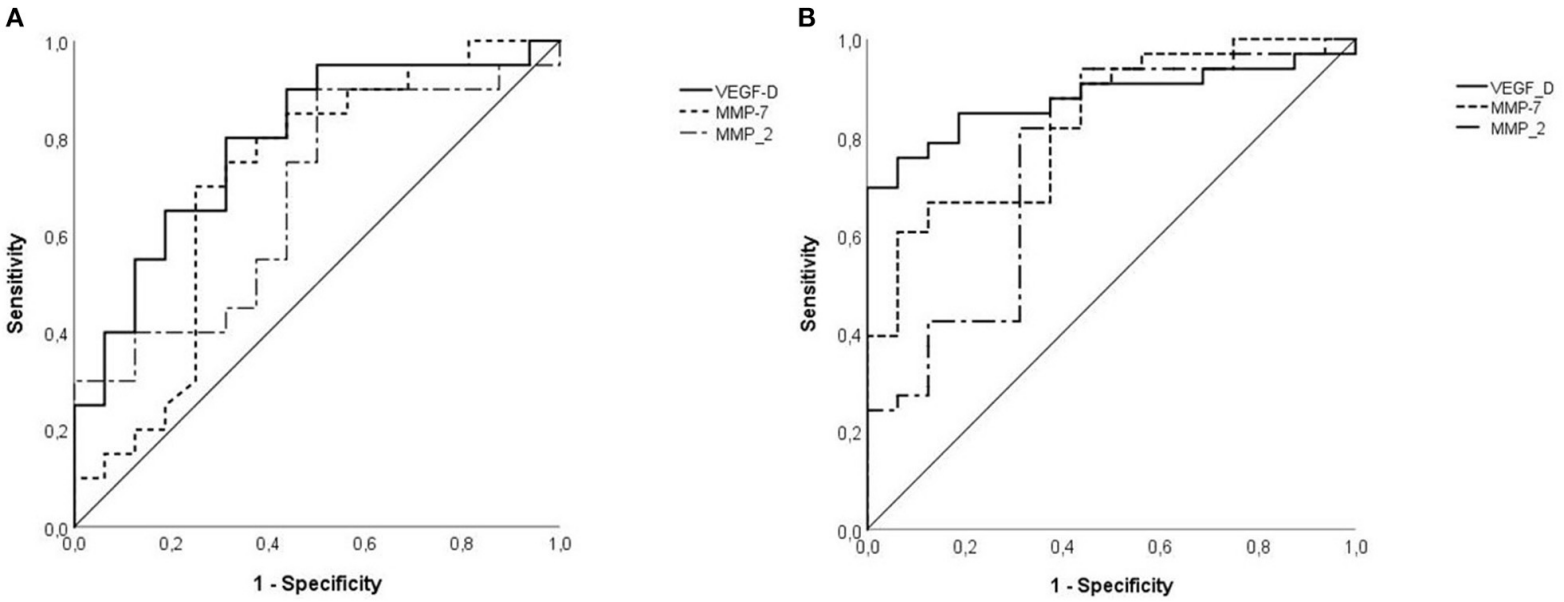
**(A)** VEGF-D was an effective diagnostic test to predict LAM [area under curve (AUC): 0.879 ± 0.049 (95% CI: 0.782–0.975), *p* < 0.001] continuous line, respect to MMP2 [AUC: 0.756 ± 0.079 (95% CI: 0.601–0.910)], dotted line, and MMP7 [0.828 ± 0.060 (95% CI: 0.710–0.945), *p* < 0.001], punctuate line. **(B)** Specificity of VEGF-D for LAM disease in TSC patients was lower than in previous analysis but remains significant [AUC: 0.791 ± 0.077 (95% CI: 0.640–0.941), *p* = 0.003], continuous line. MMP-2 has lower accuracy than VEGF-D with an AUC of 0.694 ± 0.088 (95% CI: 0.521–0.867), *p* = 0.044, dotted line and similarly MMP-7 has an AUC of 0.713 ± 0.090 (95% CI: 0.538–0.889), *p* = 0.027, punctuate line.

The area under ROC curves (AUC) exploring the ability of MMP-2 to predict LAM diagnosis was 0.756 ± 0.079 (95% CI: 0.601–0.910, *p* = 0.004). At a cut-off level of 263 pg/ml MMP-2 showed a sensitivity for LAM diagnosis of 81%, and a specificity of 69% (Figure 2A). Serum level of MMP2 ≥463 ng/ml showed a specificity for LAM diagnosis of 100%. Only nine patients had MMP-2 serum level higher than 463 ng/ml, with a sensitivity of 24%. MMP-7 resulted a better biomarker for the diagnosis of LAM than MMP-2 with an AUC of 0.828 ± 0.060 (95% CI: 0.710–0.945, *p* < 0.001). The optimal cut off value resulted as 3.27 pg/ml, with a sensitivity and specificity of 67 and 82%, respectively (Figure 2A). Serum level of MMP-7 ≥4.8 pg/ml showed a specificity for LAM diagnosis of 100%. Fourteen patients had MMP-7 serum level higher than 4.8 pg/ml, with a sensitivity of 40%.

The cut off value of 800 pg/mL for VEGF-D had sensitivity and specificity for the diagnosis of LAM of 58 and 100%, respectively The ROC AUC for the diagnosis of LAM of VEGF-D was 0.879 ± 0.049 (95% CI: 0.782–0.975, *p* < 0.001, Figure 2A).

### Diagnostic Yield of Biomarkers for LAM in TSC Patients

Considering all patients with TSC, the AUC for MMP-2 in predicting LAM was 0.694 ± 0.088 (95% CI: 0.521–0.867, *p* = 0.044, Figure 2B). With a cut off value of 340 pg/dL, the specificity was 88%, and the sensitivity was 40%. Serum level of MMP-2 ≥461 pg/ml showed a specificity for LAM diagnosis of 100%. Seven patients had MMP-2 serum level higher than 461 pg/ml, with a sensitivity of 30%. The diagnostic yield of MMP-7 for LAM diagnosis was similar to MMP-2, with an AUC of 0.713 ± 0.090 (95% CI: 0.538–0.889, *p* = 0.027, Figure 2B). 4.0 pg/mL was the optimal cut off value for LAM with a sensitivity and a specificity of 75 and 71%, respectively. Serum level of MMP-7 ≥8.73 ng/mL showed a sensitivity of 100%. Three patients had serum level of MMP-7 higher than 8.73 ng/mL, with a sensitivity of 10%. In patients with TSC the threshold of 800 pg/mL for VEGF-D had a sensitivity of 55% and a specificity of 82%. The AUC for LAM diagnosis was 0.791 ± 0.077 (95% CI: 0.640–0.941, *p* = 0.003, Figure 2B).

## Discussion

The most important results of this study are: (1) serum level of MMP-2 is significantly elevated in patients with S-LAM and TSC-LAM compared to patients with TSC and controls, while serum MMP-7 is higher in patients with TSC-LAM than in patients with S-LAM and in controls; (2) patients with minimal pulmonary disease seem to have higher levels of MMP-7 and MMP-2; whether this is due a more active disease and LAM-cell migration needs further studies; (3) high levels of MMP-2 or MMP-7 are not associated with a more severe systemic involvement; (4) high VEGF-D seems to be associated with younger age, *TSC2* mutational status, and more severe systemic involvement; (5) VEGF-C does not seem to have a role as biomarker in LAM and TSC.

The MMPs and their tissue inhibitors *in vivo* are involved in remodeling the extracellular matrix and basement membranes both in normal and pathologic conditions (35). Overespression of MMPs was correlated with a more aggressive phenotype of cancer (36); the overexpression of MMP-7 is associated with tumor proliferation and a poor prognosis in some pulmonary disorders such as non-small cell lung carcinoma and idiopathic pulmonary fibrosis (36, 37). Some immunohistochemical studied have demonstrated that MMP-2 and their tissue inhibitors are over expressed in the pulmonary tissue of patients with LAM compared to normal bronchial tissue (18, 38). MMPs are highly susceptible to a variety of regulators including hormones, pro-inflammatory cytokines, and growth factors. Although the subtypes of the MMP family are not abundant in the healthy lung, they are produced by inflammatory cells and lungs in response to inflammatory chemokines and free oxygen radicals (39). In cancer, interleukins (ILs) induce neo-angiogenesis and enhance the activity of MMPs, which increases the metastatic activity as demonstrated in hepatocellular carcinoma where IL-8 and IL-17 cause MMP-2 and MMP-9 activation (40). MMPs may likely be the result or the cause of a condition, as demonstrated by the increased expression of MMP-7 in the bronchial epithelial cells of asthmatic patients, and, conversely, the association of MMP-7 with wound repair and low expression in asthmatic patients (41). Natural inhibitors such as tissue inhibitor of TIMPs, protease inhibitor homologs known as TIMP-1, TIMP-2, TIMP-3, and TIMP-4, also regulate MMPs activity (42). The imbalance between MMPs and TIMPs, together with the increased levels of MMPs, seems to cause the cystic destruction of lung LAM parenchyma (43).

Several agents can target the MMPs to inhibit MMPs activation, MMPs enzyme activity, and to suppress MMPs at the transcription/translation levels. Among these we can list natural products such as Neovastat and curcumin, the broad-spectrum antibiotics tetracyclines, bisphosphonates generally used to treat osteoporosis and bone diseases, and histone deacetylase inhibitors (HDACIs) including vorinostat and valproic acid (44–46). Various antiepileptic drugs (AEDs) such as phenytoin (47), valproic acid and lamotrigine (48) have also been demonstrated to exert an inhibitory effect on MMPs. Since epilepsy is a common manifestation of TSC, MMPs expression should be evaluated taking into account whether the patient is taking any AEDs and, in such case, which one. However, in our study, serum MMP-7 level was higher in TSC-LAM patients than in healthy individuals and MMP-2 was increased in S-LAM and TSC-LAM patients compared to TSC patients and controls. Moreover, the low number of patients for each group limits the statistical analysis of the possible antiepileptic drug administration related to MMPs levels.

Lee et al. demonstrated that cells lacking the *TSC1/TSC2* genes overexpressed MMP-2 and that this overexpression was not affected by rapamycin (49). Our data indicate that the MMP-2 serum levels are higher in patients with LAM than in patients without LAM, but we also observed a high overlap between subgroups of the single values. These data are consistent with previous studies on this field. Moses et al. described a patient with LAM in whom urinary levels of some MMPs isoforms (in particular MMP-2 and−9) were elevated and decreased after treatment with doxycycline (an MMP inhibitor) (50). Pimenta et al. described a cohort of 41 patients also treated with doxycycline. Serum and urinary levels of MMP-2 were higher in patients with LAM then in healthy controls and decreased after treatment with the antibiotic; however, the median of MMP-2 in serum was below the detection limit both at baseline and after treatment (50, 51). Chang and colleagues analyzed some serum biomarkers as diagnostic and prognostic tools, and found higher MMP-2 levels in patients with LAM than in controls with a considerable overlap of single values between the two groups (52). Finally, Odajima et al. studied in 2009 the serum level of MMP-2 and MMP-9 in 36 patients with LAM and did not find any significant differences from healthy controls (21). In this study the ROC analysis have furthermore demonstrated that the ability of MMP-2 to predict LAM disease was lower than the ability of VEGF-D in line with the previously cited work by Chang et al. (52). The previous demonstration that MMP-7 and β-catenin are expressed in LAM tissues suggests that tuberin-deficient cells acquire invasive characteristics which may underlie the development of LAM disease (24). Interestingly, the serum MMP-7 levels were high in all the LAM/TSC patients, while in S-LAM and TSC patients MMP-7 show similar levels of healthy subjects. To understand this data further analysis are needed, likely keeping in consideration the significant relationship of high MMP-7 levels with the higher frequency of cortical tubers.

VEGF-D serum levels were analyzed in previous studies that led this biomarker to be included in the recent diagnostic guidelines as a diagnostic tool in the presence of a typical chest CT pattern, thus reducing the need for lung biopsy in patients with suspected LAM (10). In two studies developed by Seyama et al. and Glasgow et al., VEGF-D serum levels were significantly higher in LAM patients that in controls (12, 53). In 2013 Xu et al. found similar results with serum VEGF-D levels significantly increased in the definite LAM group, compared with that of healthy controls (11). Young et al. measured serum level of VEGF-D in patients with LAM, healthy controls and patients with other pulmonary diseases and found that serum levels of VEGF-D were significantly higher in the first group of patients (9). Similarly, Radzikowska et al. showed that VEGF-D could discriminate between LAM and other pulmonary cystic diseases such as pulmonary Langerhans cell histiocytosis and lymphocytic interstitial pneumonia (54). In line with this data, our results demonstrate higher VEGF-D serum levels in LAM patients than in healthy controls. Nevertheless, in our study group more than 40% of patients with a definite diagnosis of LAM showed serum levels of VEGF-D lower than the diagnostic threshold of 800 pg/mL; VEGF-D has a sensitivity of 58% and a specificity of 100% for the diagnosis of LAM. VEGF-D high specificity is confirmed with a low sensitivity. This is consistent with the study by Chang in which 42% of patients with LAM showed VEGF-D serum levels lower than the diagnostic threshold and a sensitivity of 56% and a specificity of 100% (52). On the contrary, Xu et al. found a VEGF-D sensitivity of 96% (11). In a study from Glasgow et al., a statistically significant difference between LAM and healthy controls for VEGF-D serum level was maintained only for LAM patients with lymphatic involvement (lymphangioleiomyomas and/or lymphadenopathy) but not for those patients with a disease restricted to the lungs (12). In our analysis, however, we did not find any difference in lymphatic involvement in patients with a VEGF-D higher or lower than the diagnostic threshold of 800 pg/mL except for chylothorax, more frequent in patients with a VEGF-D higher than 800 pg/mL. However, it is possible that these results may be related to some differences in the studied population. The differences in the number of patients involved in the studies could in part explain these discrepancies and affect the statistical significance. Furthermore, in our cohort there was a low percentage of lymphatic involvement.

We found a trend to higher VEGF-D serum levels in patients with TSC-LAM compared to patients with TSC and a normal high-resolution CT scan. This is in line with data published by Young et al. in 2008, where VEGF-D levels were much higher in women with TSC and LAM than in women with TSC and normal high-resolution CT scan (9). However, the authors found a very strong difference between the two groups in contrast to our data that show only a trend to statistical significance. This difference may be ascribed to some differences in the studied population as well. In fact, the majority of our patients with TSC-LAM had a mild disease, while we do not have data on the population analyzed in Young's study. Furthermore, TSC is a disease with a very heterogeneous presentation and systemic involvement, and the extent of lymphatic involvement could have influenced the analysis. Our data indicate that patients with VEGF-D serum levels above the diagnostic threshold of 800 pg/ml show more frequently pathogenic variant in *TSC2*. This is consistent with previous studies showing that there is a higher rate of *TSC2* mutations than *TSC1* mutations in patients with TSC-LAM (55–57) and that patients with TSC and pathogenic variants in *TSC1* usually have a milder disease in comparison with patients carrying *TSC2* pathogenic variants (58).

This work has two innovative characteristics: first, we performed deep phenotyping of the whole population analyzing separately and comparing the serum levels of four biomarkers in patients with S-LAM and TSC-LAM and exploring a possible link with clinical and genetic characteristics of the single groups. Secondly, this is the first study that has analyzed serum levels of MMP-7 in relationship to LAM. However, some potential limits to the present study deserve discussion. The main limitation of our study is the limited number of patients included, because of the rarity of the disease and the monocentric nature of the study. We enrolled patients with a definite diagnosis of TSC and LAM and healthy individuals. In clinical practice, the diagnostic challenge is represented by the patients with a cystic lung disease. In keeping with the explorative nature of the study we tried to evaluate the serum biomarkers level in a “pure” population to investigate the data distribution (narrow distribution or very scattered data). Further studies involving other cystic lung diseases as controls (i.e., Birt Hogg Dubè Syndrome, lymphoid interstitial pneumonia, Langherans cells Histiocytosis) and with multiple samplings are needed to strengthen the specificity of the biomarkers for LAM and to evaluate data reproducibility over time and changes in case of treatment or recurrence.

Finally, enrolled patients show a relatively mild disease, in terms of pulmonary function and the clinical and radiological data used to explore a possible link between single biomarkers and systemic involvement were requested for clinical follow up purpose, and some data are therefore missing due to the “retrospective” nature of the analysis.

In conclusion, MMP-2 and especially MMP-7 are promising biomarkers for LAM and validation in longitudinal studies and with a larger patient population is needed. The diagnostic value of VEGF-D for LAM was confirmed in this cohort of Italian patients.

## Data Availability Statement

The raw data supporting the conclusions of this article will be made available by the authors, without undue reservation.

## Ethics Statement

The studies involving human participants were reviewed and approved by Comitato Etico Interaziendale Milano Area A. The patients/participants provided their written informed consent to participate in this study.

## Author Contributions

STe, FD, and EL had full access to all of the data in the study and takes responsibility for the integrity of the data, the accuracy of the data analysis, study concept, and design. STe, SA, GI, LG, GP, PC, and STr: acquisition of data. STe, FD, and GI: analysis and interpretation of data. STe and FD: drafting of the manuscript. OT, AP, SC, and EL: critical revision of the manuscript for important intellectual content. STe and GI: statistical analysis. All authors reviewed and approved the final version of the manuscript.

## Conflict of Interest

The authors declare that the research was conducted in the absence of any commercial or financial relationships that could be construed as a potential conflict of interest.
